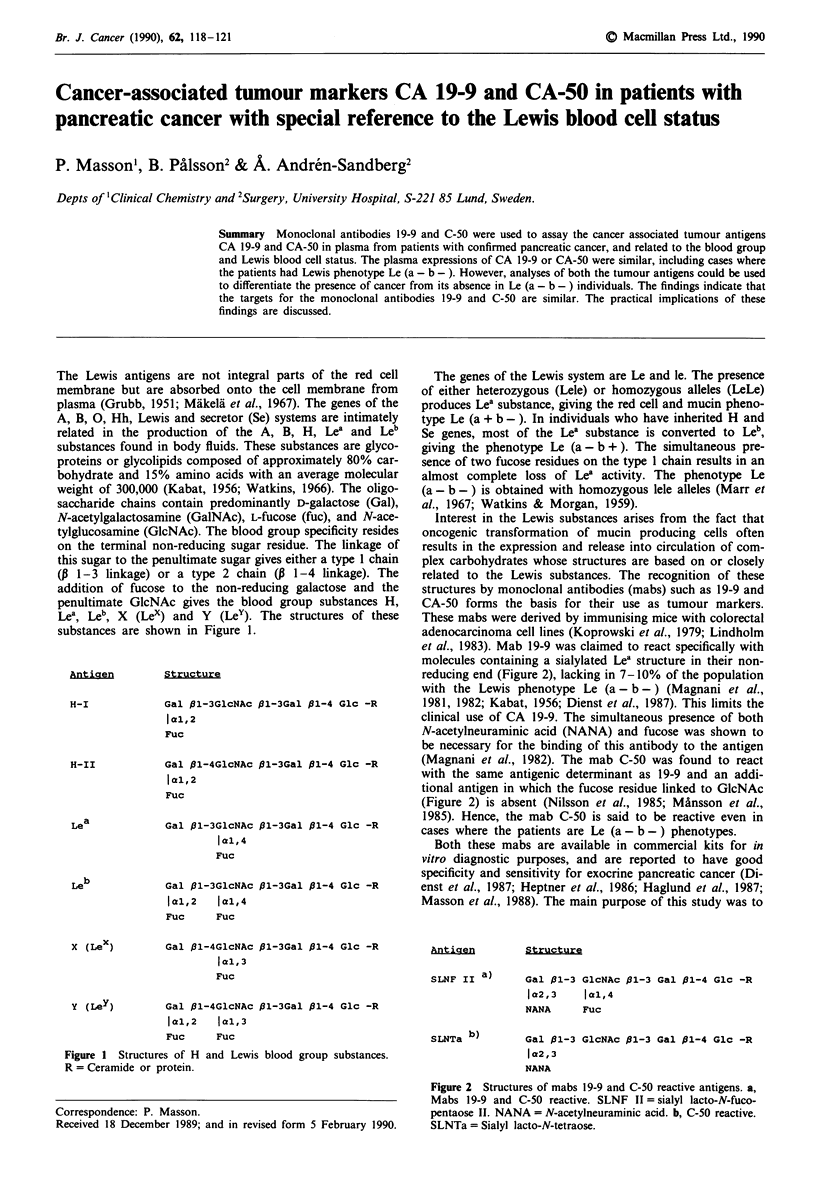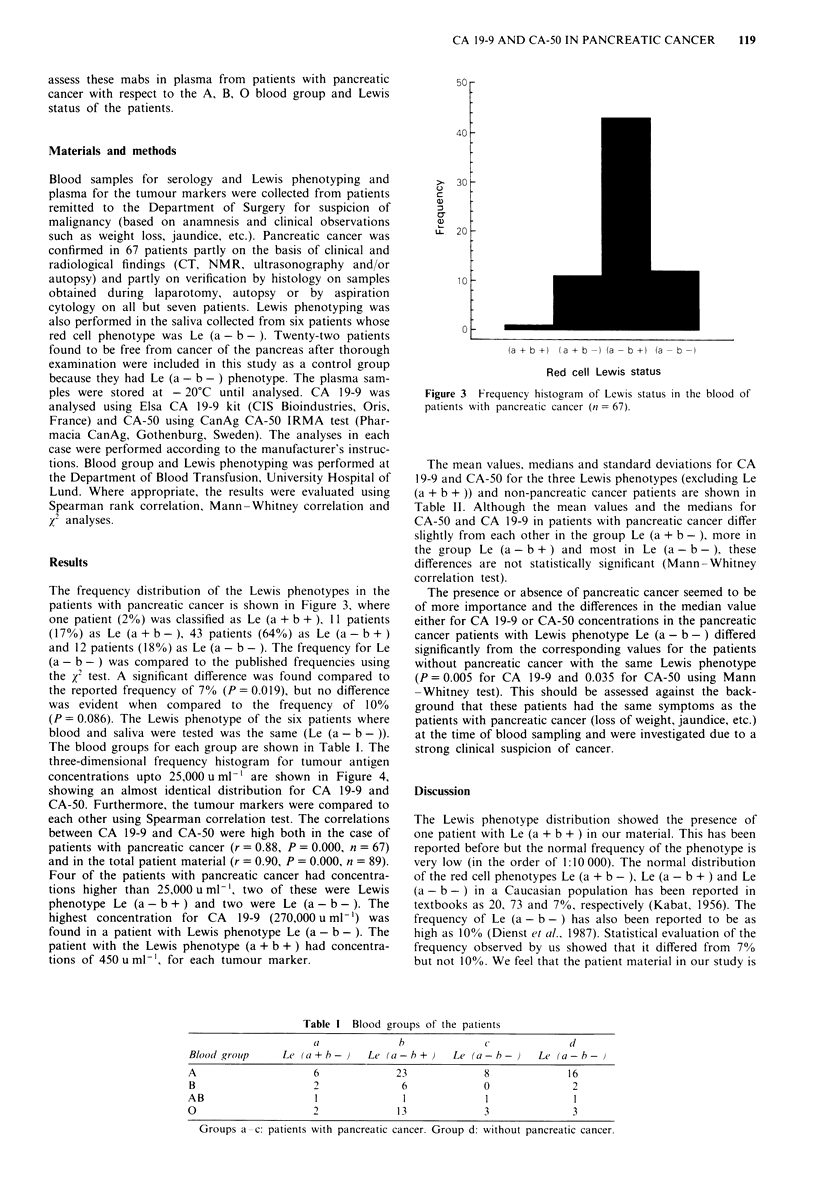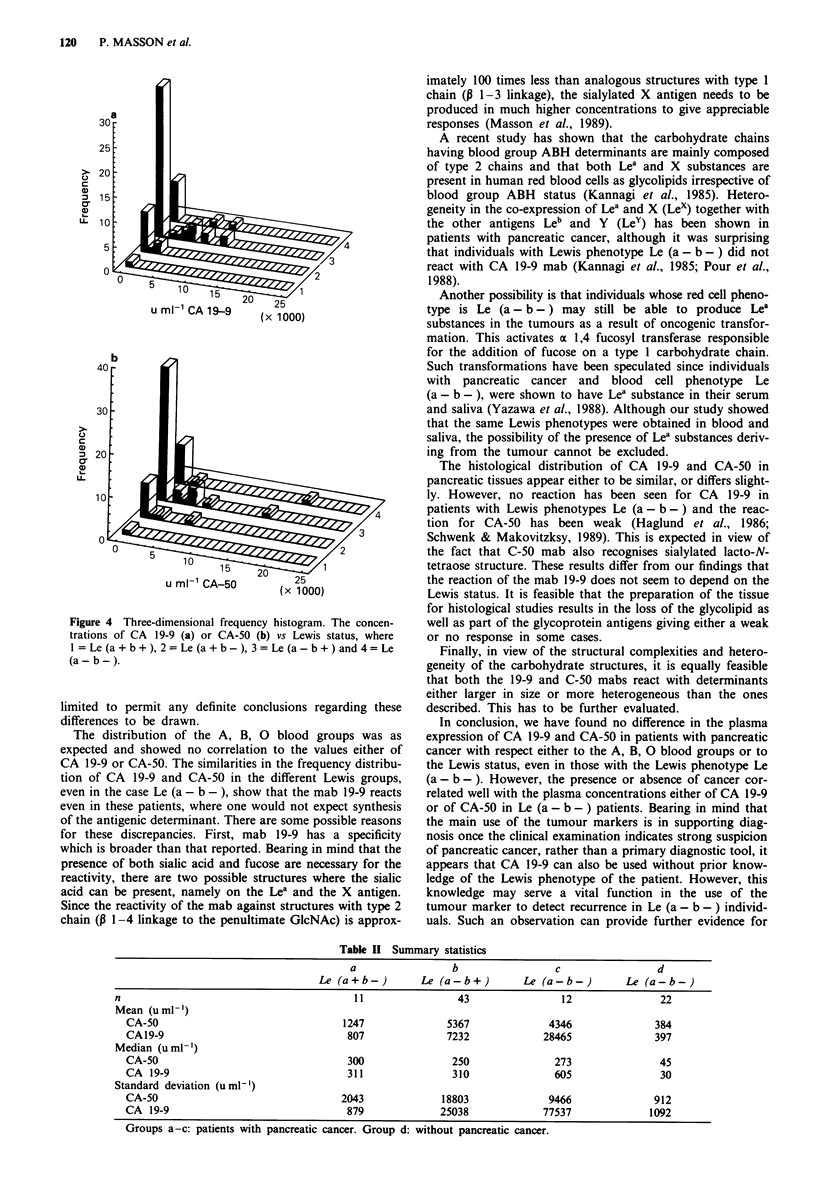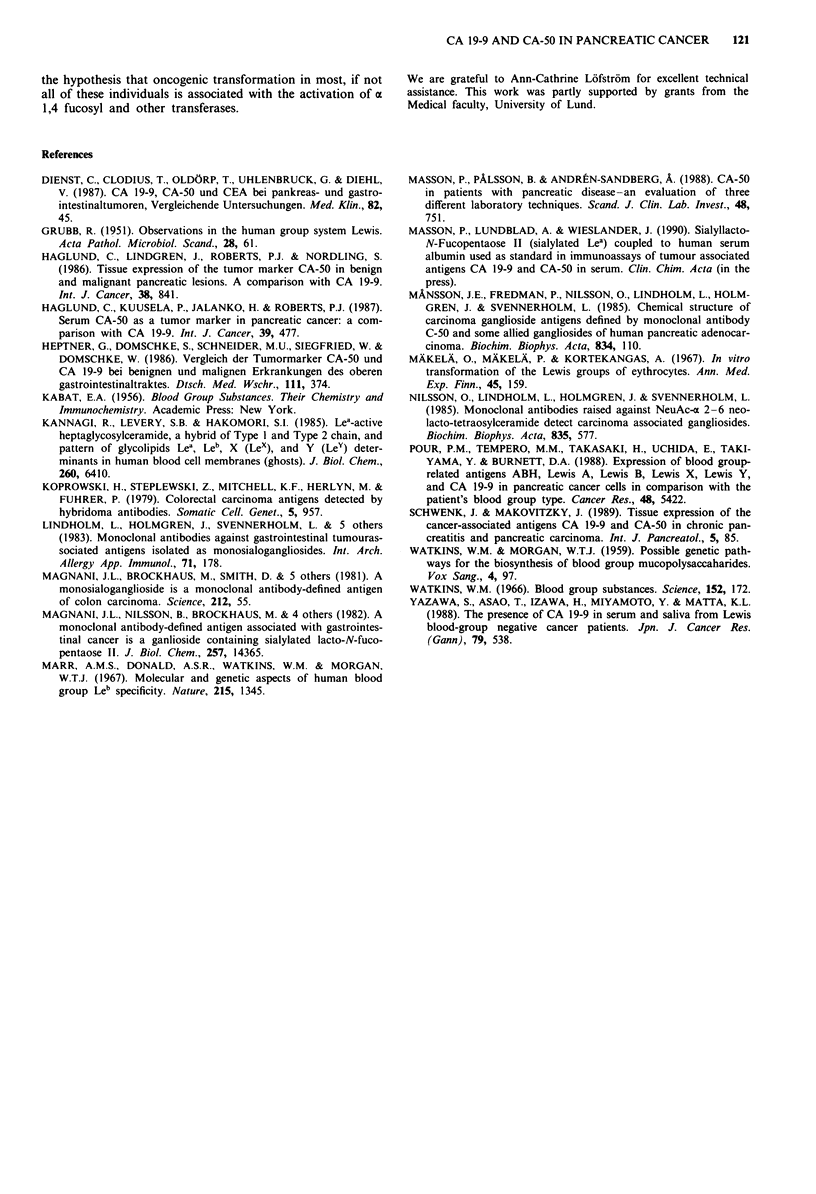# Cancer-associated tumour markers CA 19-9 and CA-50 in patients with pancreatic cancer with special reference to the Lewis blood cell status.

**DOI:** 10.1038/bjc.1990.241

**Published:** 1990-07

**Authors:** P. Masson, B. Pålsson, A. Andrén-Sandberg

**Affiliations:** Dept of Clinical Chemistry, University Hospital, Lund, Sweden.

## Abstract

Monoclonal antibodies 19-9 and C-50 were used to assay the cancer associated tumour antigens CA 19-9 and CA-50 in plasma from patients with confirmed pancreatic cancer, and related to the blood group and Lewis blood cell status. The plasma expressions of CA 19-9 or CA-50 were similar, including cases where the patients had Lewis phenotype Le (a-b-). However, analyses of both the tumour antigens could be used to differentiate the presence of cancer from its absence in Le (a-b-) individuals. The findings indicate that the targets for the monoclonal antibodies 19-9 and C-50 are similar. The practical implications of these findings are discussed.


Br. J. Cancer (1990), 62, 118-121 © Macmillan Press Ltd., 1990

### Cancer-associated tumour markers CA 19-9 and CA-50 in patients with pancreatic cancer with special reference to the Lewis blood cell status

P. Masson[1], B. Pålsson[2] & Å. Andrén-Sandberg[2]

*Depts of [1]Clinical Chemistry and [2]Surgery, University Hospital, S-221 85 Lund, Sweden.*

**Summary** Monoclonal antibodies 19-9 and C-50 were used to assay the cancer associated tumour antigens CA 19-9 and CA-50 in plasma from patients with confirmed pancreatic cancer, and related to the blood group and Lewis blood cell status. The plasma expressions of CA 19-9 or CA-50 were similar, including cases where the patients had Lewis phenotype Le (a − b − ). However, analyses of both the tumour antigens could be used to differentiate the presence of cancer from its absence in Le (a − b − ) individuals. The findings indicate that the targets for the monoclonal antibodies 19-9 and C-50 are similar. The practical implications of these findings are discussed.

The Lewis antigens are not integral parts of the red cell membrane but are absorbed onto the cell membrane from plasma (Grubb, 1951; Mäkelä *et al.*, 1967). The genes of the A, B, O, Hh, Lewis and secretor (Se) systems are intimately related in the production of the A, B, H, Le$^a$ and Le$^b$ substances found in body fluids. These substances are glycoproteins or glycolipids composed of approximately 80% carbohydrate and 15% amino acids with an average molecular weight of 300,000 (Kabat, 1956; Watkins, 1966). The oligosaccharide chains contain predominantly D-galactose (Gal), *N*-acetylgalactosamine (GalNAc), L-fucose (fuc), and *N*-acetylglucosamine (GlcNAc). The blood group specificity resides on the terminal non-reducing sugar residue. The linkage of this sugar to the penultimate sugar gives either a type 1 chain (β 1−3 linkage) or a type 2 chain (β 1−4 linkage). The addition of fucose to the non-reducing galactose and the penultimate GlcNAc gives the blood group substances H, Le$^a$, Le$^b$, X (Le$^X$) and Y (Le$^Y$). The structures of these substances are shown in Figure 1.

```

Antigen     Structure

H-I         Gal β1-3GlcNAc β1-3Gal β1-4 Glc -R
            |α1,2
            Fuc

H-II        Gal β1-4GlcNAc β1-3Gal β1-4 Glc -R
            |α1,2
            Fuc

Le^a        Gal β1-3GlcNAc β1-3Gal β1-4 Glc -R
                |α1,4
                Fuc

Le^b        Gal β1-3GlcNAc β1-3Gal β1-4 Glc -R
            |α1,2   |α1,4
            Fuc     Fuc

X (Le^X)    Gal β1-4GlcNAc β1-3Gal β1-4 Glc -R
                |α1,3
                Fuc

Y (Le^Y)    Gal β1-4GlcNAc β1-3Gal β1-4 Glc -R
            |α1,2   |α1,3
            Fuc     Fuc
```

**Figure 1** Structures of H and Lewis blood group substances. R = Ceramide or protein.

Correspondence: P. Masson.
Received 18 December 1989; and in revised form 5 February 1990.

The genes of the Lewis system are Le and le. The presence of either heterozygous (Lele) or homozygous alleles (LeLe) produces Le$^a$ substance, giving the red cell and mucin phenotype Le (a + b − ). In individuals who have inherited H and Se genes, most of the Le$^a$ substance is converted to Le$^b$, giving the phenotype Le (a − b + ). The simultaneous presence of two fucose residues on the type 1 chain results in an almost complete loss of Le$^a$ activity. The phenotype Le (a − b − ) is obtained with homozygous lele alleles (Marr *et al.*, 1967; Watkins & Morgan, 1959).

Interest in the Lewis substances arises from the fact that oncogenic transformation of mucin producing cells often results in the expression and release into circulation of complex carbohydrates whose structures are based on or closely related to the Lewis substances. The recognition of these structures by monoclonal antibodies (mabs) such as 19-9 and CA-50 forms the basis for their use as tumour markers. These mabs were derived by immunising mice with colorectal adenocarcinoma cell lines (Koprowski *et al.*, 1979; Lindholm *et al.*, 1983). Mab 19-9 was claimed to react specifically with molecules containing a sialylated Le$^a$ structure in their non-reducing end (Figure 2), lacking in 7−10% of the population with the Lewis phenotype Le (a − b − ) (Magnani *et al.*, 1981, 1982; Kabat, 1956; Dienst *et al.*, 1987). This limits the clinical use of CA 19-9. The simultaneous presence of both *N*-acetylneuraminic acid (NANA) and fucose was shown to be necessary for the binding of this antibody to the antigen (Magnani *et al.*, 1982). The mab C-50 was found to react with the same antigenic determinant as 19-9 and an additional antigen in which the fucose residue linked to GlcNAc (Figure 2) is absent (Nilsson *et al.*, 1985; Månsson *et al.*, 1985). Hence, the mab C-50 is said to be reactive even in cases where the patients are Le (a − b − ) phenotypes.

Both these mabs are available in commercial kits for *in vitro* diagnostic purposes, and are reported to have good specificity and sensitivity for exocrine pancreatic cancer (Dienst *et al.*, 1987; Heptner *et al.*, 1986; Haglund *et al.*, 1987; Masson *et al.*, 1988). The main purpose of this study was to

```

Antigen       Structure

SLNF II a)    Gal β1-3 GlcNAc β1-3 Gal β1-4 Glc -R
              |α2,3     |α1,4
              NANA      Fuc

SLNTa b)      Gal β1-3 GlcNAc β1-3 Gal β1-4 Glc -R
              |α2,3
              NANA
```

**Figure 2** Structures of mabs 19-9 and C-50 reactive antigens. **a**, Mabs 19-9 and C-50 reactive. SLNF II = sialyl lacto-*N*-fucopentaose II. NANA = *N*-acetylneuraminic acid. **b**, C-50 reactive. SLNTa = Sialyl lacto-*N*-tetraose.

assess these mabs in plasma from patients with pancreatic cancer with respect to the A, B, O blood group and Lewis status of the patients.

### Materials and methods

Blood samples for serology and Lewis phenotyping and plasma for the tumour markers were collected from patients remitted to the Department of Surgery for suspicion of malignancy (based on anamnesis and clinical observations such as weight loss, jaundice, etc.). Pancreatic cancer was confirmed in 67 patients partly on the basis of clinical and radiological findings (CT, NMR, ultrasonography and/or autopsy) and partly on verification by histology on samples obtained during laparotomy, autopsy or by aspiration cytology on all but seven patients. Lewis phenotyping was also performed in the saliva collected from six patients whose red cell phenotype was Le (a – b – ). Twenty-two patients found to be free from cancer of the pancreas after thorough examination were included in this study as a control group because they had Le (a – b – ) phenotype. The plasma samples were stored at – 20°C until analysed. CA 19-9 was analysed using Elsa CA 19-9 kit (CIS Bioindustries, Oris, France) and CA-50 using CanAg CA-50 IRMA test (Pharmacia CanAg, Gothenburg, Sweden). The analyses in each case were performed according to the manufacturer's instructions. Blood group and Lewis phenotyping was performed at the Department of Blood Transfusion, University Hospital of Lund. Where appropriate, the results were evaluated using Spearman rank correlation, Mann–Whitney correlation and $\chi^2$ analyses.

### Results

The frequency distribution of the Lewis phenotypes in the patients with pancreatic cancer is shown in Figure 3, where one patient (2%) was classified as Le (a + b + ), 11 patients (17%) as Le (a + b – ), 43 patients (64%) as Le (a – b + ) and 12 patients (18%) as Le (a – b – ). The frequency for Le (a – b – ) was compared to the published frequencies using the $\chi^2$ test. A significant difference was found compared to the reported frequency of 7% ($P = 0.019$), but no difference was evident when compared to the frequency of 10% ($P = 0.086$). The Lewis phenotype of the six patients where blood and saliva were tested was the same (Le (a – b – )). The blood groups for each group are shown in Table I. The three-dimensional frequency histogram for tumour antigen concentrations upto 25,000 u ml$^{-1}$ are shown in Figure 4, showing an almost identical distribution for CA 19-9 and CA-50. Furthermore, the tumour markers were compared to each other using Spearman correlation test. The correlations between CA 19-9 and CA-50 were high both in the case of patients with pancreatic cancer ($r = 0.88$, $P = 0.000$, $n = 67$) and in the total patient material ($r = 0.90$, $P = 0.000$, $n = 89$). Four of the patients with pancreatic cancer had concentrations higher than 25,000 u ml$^{-1}$, two of these were Lewis phenotype Le (a – b + ) and two were Le (a – b – ). The highest concentration for CA 19-9 (270,000 u ml$^{-1}$) was found in a patient with Lewis phenotype Le (a – b – ). The patient with the Lewis phenotype (a + b + ) had concentrations of 450 u ml$^{-1}$, for each tumour marker.

**Figure 3** Frequency histogram of Lewis status in the blood of patients with pancreatic cancer ($n = 67$).

The mean values, medians and standard deviations for CA 19-9 and CA-50 for the three Lewis phenotypes (excluding Le (a + b + )) and non-pancreatic cancer patients are shown in Table II. Although the mean values and the medians for CA-50 and CA 19-9 in patients with pancreatic cancer differ slightly from each other in the group Le (a + b – ), more in the group Le (a – b + ) and most in Le (a – b – ), these differences are not statistically significant (Mann–Whitney correlation test).

The presence or absence of pancreatic cancer seemed to be of more importance and the differences in the median value either for CA 19-9 or CA-50 concentrations in the pancreatic cancer patients with Lewis phenotype Le (a – b – ) differed significantly from the corresponding values for the patients without pancreatic cancer with the same Lewis phenotype ($P = 0.005$ for CA 19-9 and 0.035 for CA-50 using Mann–Whitney test). This should be assessed against the background that these patients had the same symptoms as the patients with pancreatic cancer (loss of weight, jaundice, etc.) at the time of blood sampling and were investigated due to a strong clinical suspicion of cancer.

### Discussion

The Lewis phenotype distribution showed the presence of one patient with Le (a + b + ) in our material. This has been reported before but the normal frequency of the phenotype is very low (in the order of 1:10 000). The normal distribution of the red cell phenotypes Le (a + b – ), Le (a – b + ) and Le (a – b – ) in a Caucasian population has been reported in textbooks as 20, 73 and 7%, respectively (Kabat, 1956). The frequency of Le (a – b – ) has also been reported to be as high as 10% (Dienst *et al.*, 1987). Statistical evaluation of the frequency observed by us showed that it differed from 7% but not 10%. We feel that the patient material in our study is

**Table I** Blood groups of the patients

| *Blood group* | *a* *Le (a + b – )* | *b* *Le (a – b + )* | *c* *Le (a – b – )* | *d* *Le (a – b – )* |
|---|---|---|---|---|
| A | 6 | 23 | 8 | 16 |
| B | 2 | 6 | 0 | 2 |
| AB | 1 | 1 | 1 | 1 |
| O | 2 | 13 | 3 | 3 |

Groups a–c: patients with pancreatic cancer. Group d: without pancreatic cancer.

**Figure 4** Three-dimensional frequency histogram. The concentrations of CA 19-9 (**a**) or CA-50 (**b**) *vs* Lewis status, where 1 = Le (a + b + ), 2 = Le (a + b − ), 3 = Le (a − b + ) and 4 = Le (a − b − ).

limited to permit any definite conclusions regarding these differences to be drawn.

The distribution of the A, B, O blood groups was as expected and showed no correlation to the values either of CA 19-9 or CA-50. The similarities in the frequency distribution of CA 19-9 and CA-50 in the different Lewis groups, even in the case Le (a − b − ), show that the mab 19-9 reacts even in these patients, where one would not expect synthesis of the antigenic determinant. There are some possible reasons for these discrepancies. First, mab 19-9 has a specificity which is broader than that reported. Bearing in mind that the presence of both sialic acid and fucose are necessary for the reactivity, there are two possible structures where the sialic acid can be present, namely on the $Le^a$ and the X antigen. Since the reactivity of the mab against structures with type 2 chain (β 1–4 linkage to the penultimate GlcNAc) is approximately 100 times less than analogous structures with type 1 chain (β 1–3 linkage), the sialylated X antigen needs to be produced in much higher concentrations to give appreciable responses (Masson *et al*., 1989).

A recent study has shown that the carbohydrate chains having blood group ABH determinants are mainly composed of type 2 chains and that both $Le^a$ and X substances are present in human red blood cells as glycolipids irrespective of blood group ABH status (Kannagi *et al*., 1985). Heterogeneity in the co-expression of $Le^a$ and X ($Le^X$) together with the other antigens $Le^b$ and Y ($Le^Y$) has been shown in patients with pancreatic cancer, although it was surprising that individuals with Lewis phenotype Le (a − b − ) did not react with CA 19-9 mab (Kannagi *et al*., 1985; Pour *et al*., 1988).

Another possibility is that individuals whose red cell phenotype is Le (a − b − ) may still be able to produce $Le^a$ substances in the tumours as a result of oncogenic transformation. This activates α 1,4 fucosyl transferase responsible for the addition of fucose on a type 1 carbohydrate chain. Such transformations have been speculated since individuals with pancreatic cancer and blood cell phenotype Le (a − b − ), were shown to have $Le^a$ substance in their serum and saliva (Yazawa *et al*., 1988). Although our study showed that the same Lewis phenotypes were obtained in blood and saliva, the possibility of the presence of $Le^a$ substances deriving from the tumour cannot be excluded.

The histological distribution of CA 19-9 and CA-50 in pancreatic tissues appear either to be similar, or differs slightly. However, no reaction has been seen for CA 19-9 in patients with Lewis phenotypes Le (a − b − ) and the reaction for CA-50 has been weak (Haglund *et al*., 1986; Schwenk & Makovitzksy, 1989). This is expected in view of the fact that C-50 mab also recognises sialylated lacto-*N*-tetraose structure. These results differ from our findings that the reaction of the mab 19-9 does not seem to depend on the Lewis status. It is feasible that the preparation of the tissue for histological studies results in the loss of the glycolipid as well as part of the glycoprotein antigens giving either a weak or no response in some cases.

Finally, in view of the structural complexities and heterogeneity of the carbohydrate structures, it is equally feasible that both the 19-9 and C-50 mabs react with determinants either larger in size or more heterogeneous than the ones described. This has to be further evaluated.

In conclusion, we have found no difference in the plasma expression of CA 19-9 and CA-50 in patients with pancreatic cancer with respect either to the A, B, O blood groups or to the Lewis status, even in those with the Lewis phenotype Le (a − b − ). However, the presence or absence of cancer correlated well with the plasma concentrations either of CA 19-9 or of CA-50 in Le (a − b − ) patients. Bearing in mind that the main use of the tumour markers is in supporting diagnosis once the clinical examination indicates strong suspicion of pancreatic cancer, rather than a primary diagnostic tool, it appears that CA 19-9 can also be used without prior knowledge of the Lewis phenotype of the patient. However, this knowledge may serve a vital function in the use of the tumour marker to detect recurrence in Le (a − b − ) individuals. Such an observation can provide further evidence for

**Table II** Summary statistics

| | *a* Le (a + b − ) | *b* Le (a − b + ) | *c* Le (a − b − ) | *d* Le (a − b − ) |
|---|---|---|---|---|
| *n* | 11 | 43 | 12 | 22 |
| Mean (u ml⁻¹) | | | | |
| CA-50 | 1247 | 5367 | 4346 | 384 |
| CA19-9 | 807 | 7232 | 28465 | 397 |
| Median (u ml⁻¹) | | | | |
| CA-50 | 300 | 250 | 273 | 45 |
| CA 19-9 | 311 | 310 | 605 | 30 |
| Standard deviation (u ml⁻¹) | | | | |
| CA-50 | 2043 | 18803 | 9466 | 912 |
| CA 19-9 | 879 | 25038 | 77537 | 1092 |

Groups a–c: patients with pancreatic cancer. Group d: without pancreatic cancer.

the hypothesis that oncogenic transformation in most, if not all of these individuals is associated with the activation of α 1,4 fucosyl and other transferases.

We are grateful to Ann-Cathrine Löfström for excellent technical assistance. This work was partly supported by grants from the Medical faculty, University of Lund.

### References

DIENST, C., CLODIUS, T., OLDÖRP, T., UHLENBRUCK, G. & DIEHL, V. (1987). CA 19-9, CA-50 und CEA bei pankreas- und gastrointestinaltumoren, Vergleichende Untersuchungen. *Med. Klin.*, **82**, 45.

GRUBB, R. (1951). Observations in the human group system Lewis. *Acta Pathol. Microbiol. Scand.*, **28**, 61.

HAGLUND, C., LINDGREN, J., ROBERTS, P.J. & NORDLING, S. (1986). Tissue expression of the tumor marker CA-50 in benign and malignant pancreatic lesions. A comparison with CA 19-9. *Int. J. Cancer*, **38**, 841.

HAGLUND, C., KUUSELA, P., JALANKO, H. & ROBERTS, P.J. (1987). Serum CA-50 as a tumor marker in pancreatic cancer: a comparison with CA 19-9. *Int. J. Cancer*, **39**, 477.

HEPTNER, G., DOMSCHKE, S., SCHNEIDER, M.U., SIEGFRIED, W. & DOMSCHKE, W. (1986). Vergleich der Tumormarker CA-50 und CA 19-9 bei benignen und malignen Erkrankungen des oberen gastrointestinaltraktes. *Dtsch. Med. Wschr.*, **111**, 374.

KABAT, E.A. (1956). *Blood Group Substances. Their Chemistry and Immunochemistry*. Academic Press: New York.

KANNAGI, R., LEVERY, S.B. & HAKOMORI, S.I. (1985). $Le^a$-active heptaglycosylceramide, a hybrid of Type 1 and Type 2 chain, and pattern of glycolipids $Le^a$, $Le^b$, X ($Le^X$), and Y ($Le^Y$) determinants in human blood cell membranes (ghosts). *J. Biol. Chem.*, **260**, 6410.

KOPROWSKI, H., STEPLEWSKI, Z., MITCHELL, K.F., HERLYN, M. & FUHRER, P. (1979). Colorectal carcinoma antigens detected by hybridoma antibodies. *Somatic Cell. Genet.*, **5**, 957.

LINDHOLM, L., HOLMGREN, J., SVENNERHOLM, L. & 5 others (1983). Monoclonal antibodies against gastrointestinal tumourassociated antigens isolated as monosialogangliosides. *Int. Arch. Allergy App. Immunol.*, **71**, 178.

MAGNANI, J.L., BROCKHAUS, M., SMITH, D. & 5 others (1981). A monosialoganglioside is a monoclonal antibody-defined antigen of colon carcinoma. *Science*, **212**, 55.

MAGNANI, J.L., NILSSON, B., BROCKHAUS, M. & 4 others (1982). A monoclonal antibody-defined antigen associated with gastrointestinal cancer is a ganlioside containing sialylated lacto-*N*-fucopentaose II. *J. Biol. Chem.*, **257**, 14365.

MARR, A.M.S., DONALD, A.S.R., WATKINS, W.M. & MORGAN, W.T.J. (1967). Molecular and genetic aspects of human blood group $Le^b$ specificity. *Nature*, **215**, 1345.

MASSON, P., PÅLSSON, B. & ANDRÉN-SANDBERG, Å. (1988). CA-50 in patients with pancreatic disease–an evaluation of three different laboratory techniques. *Scand. J. Clin. Lab. Invest.*, **48**, 751.

MASSON, P., LUNDBLAD, A. & WIESLANDER, J. (1990). Sialyllacto-*N*-Fucopentaose II (sialylated $Le^a$) coupled to human serum albumin used as standard in immunoassays of tumour associated antigens CA 19-9 and CA-50 in serum. *Clin. Chim. Acta* (in the press).

MÅNSSON, J.E., FREDMAN, P., NILSSON, O., LINDHOLM, L., HOLMGREN, J. & SVENNERHOLM, L. (1985). Chemical structure of carcinoma ganglioside antigens defined by monoclonal antibody C-50 and some allied gangliosides of human pancreatic adenocarcinoma. *Biochim. Biophys. Acta*, **834**, 110.

MÄKELÄ, O., MÄKELÄ, P. & KORTEKANGAS, A. (1967). *In vitro* transformation of the Lewis groups of eythrocytes. *Ann. Med. Exp. Finn.*, **45**, 159.

NILSSON, O., LINDHOLM, L., HOLMGREN, J. & SVENNERHOLM, L. (1985). Monoclonal antibodies raised against NeuAc-α 2-6 neolacto-tetraosylceramide detect carcinoma associated gangliosides. *Biochim. Biophys. Acta*, **835**, 577.

POUR, P.M., TEMPERO, M.M., TAKASAKI, H., UCHIDA, E., TAKIYAMA, Y. & BURNETT, D.A. (1988). Expression of blood group-related antigens ABH, Lewis A, Lewis B, Lewis X, Lewis Y, and CA 19-9 in pancreatic cancer cells in comparison with the patient's blood group type. *Cancer Res.*, **48**, 5422.

SCHWENK, J. & MAKOVITZKY, J. (1989). Tissue expression of the cancer-associated antigens CA 19-9 and CA-50 in chronic pancreatitis and pancreatic carcinoma. *Int. J. Pancreatol.*, **5**, 85.

WATKINS, W.M. & MORGAN, W.T.J. (1959). Possible genetic pathways for the biosynthesis of blood group mucopolysaccaharides. *Vox Sang.*, **4**, 97.

WATKINS, W.M. (1966). Blood group substances. *Science*, **152**, 172.

YAZAWA, S., ASAO, T., IZAWA, H., MIYAMOTO, Y. & MATTA, K.L. (1988). The presence of CA 19-9 in serum and saliva from Lewis blood-group negative cancer patients. *Jpn. J. Cancer Res. (Gann)*, **79**, 538.